# Retro-Pharyngeal Abscess—Its Medical History and Treatment

**Published:** 1853-01

**Authors:** Charles M. Allin

**Affiliations:** Resident Surgeon of the New York Hospital.


### ARTICLE XVII.

*Retro-Pharyngeal Abscess—Its Medical History and Treatment.* By Charles M. Allin, M. D., Resident Surgeon of the New York Hospital.

The formation of abscess between the posterior wall of the pharynx and the cervical vertebræ is not of so rare occurrence as the very general science of medical and surgical authors upon the subject would prepare us to believe. When we consider that the diseases and obstructions to which the pharynx and œsophagus are liable, have received a large share of the attention of the profession, it is not a little surprising that this affection, comparatively easy in its diagnosis, in the majority of cases, and vitally important in its results, should have so generally escaped the notice of careful observers. It is very true, that in the periodical medical publications, may be found, with reference to this subject, quite a large number of valuable facts and observations, the records of the practice and opinions of thinking men, but these lose much of their value by isolation and diffusion. In but one general work,* however, upon the practice of medicine or surgery, which I have been able to examine, is there to be found more than a slight alluison to the occurrence of abscess in this situation, and, even there, we have only a citation of two cases, almost directly from the journals where they were originally published. In an addition of Sir Astley Cooper's lectures, published in London, in 1821, the sources of danger in cases of abscess are enumerated, and among them, I have found the following: "Thirdly, when not seated in parts important to life, yet by their pressure on any essential organs render the case very different. Matter, for example, seated behind the pharynx, so as to press on the trachea, will destroy life." A similar reference is made in

*"*Stokes' and Bell's Practice of Medicine*," vol. I.

another addition of the same lectures, by Mr. Tyrrell, and the occurrence of two cases briefly alluded to, the first of which being unrecognized, terminated fatally, the other recovered. In some of the medical dictionaries, too, similarly slight suggestions are met with. Mr. Porter, in an appendix to his very able work upon the "*Surgical Pathology of the Larynx and Trachea,*" states, that he has met with purulent collections between the œsophagus and trachea, and indeed Hippocrates makes a like statement, but neither of them makes any mention of such collections behind the pharynx or œsophagus.

One reason for this silence is, probably, to be derived from the fact, that other diseases with which the abscess is very generally associated, and which are, in truth, often its consequences and indices, are of so severe a character as of themselves to absorb the entire attention of the practitioner, and are attributed, without sufficiently accurate physical examination, to the influence of other and more generally recognized causes. Hence the subject is rendered one of no small degree of interest; and it will be my endeavor, in this article, to bring together as many of the facts and phenomena belonging to it as may be of practical utility, and to point out the methods by which at least relief may be afforded, and in the greater number of cases, a permanent cure be effected.

My attention was first attracted to an extended investigation of this disease by the admission of a very interesting and instructive case into the New York hospital in the early part of the summer of 1850, the phenomena and treatment of which I had the opportunity of observing during a period of nearly three months. In December of the same year, another case, very different in its origin, progress and termination, from the first, and therefore of increased interest, came under my observation; and within a little less than two years, two other cases still have been met with in the institution. In the first of these latter cases, the existence of the abscess was not detected until the post-mortem examination, the symptoms being attributed to inflammation of the larynx, and treated accordingly; and in the other, the anterior wall of the abscess was accidentally ruptured during the introduction of a probang, the object being to apply a solution of the nitrate of silver to what were supposed to be syphilitic ulcers of the throat, the true nature of the disease having been neither recognized nor suspected. In addition to these, Professor Parker, of the College of Physicians and Surgeons, has met with three patients afflicted with the disease, two of whom died, a cure being effected in the other case, after an *explorative* opening of the abscess. I have also had the opportunity to learn the history of two cases occurring in the private practice of Professor Alfred C. Post, of this city, both of which were of the chronic variety, and associated with caries of one or more of the cervical vertebræ. By the kindness of Dr. W. H. Van Buren, I have been enabled to examine a very beautiful specimen of abscess in this region, which he removed, post mortem, from the neck of a child six months old, and which is preserved in his pathological collection, in the museum attached to the College of Physicians and Surgeons. The history of the case is recorded very briefly in the minutes of the New York Pathological Society, and published in the *New York Journal of Medicine*, for July, 1850.

The earliest mention of the occurrence of abscess behind the pharynx, which I have seen, is to be found in the medical works (Praxeos Medicæ) of Platerus, published in 1625. This being included, I have encountered reports, more or less extensive, of *fifty-eight* cases; and among all these, only *twenty-eight* have been relieved or cured. This is a large mortality to attend a disease so amenable to treatment as is this, when early recognized; and there is no doubt that it is simply from the very general ignorance, or forgetfulness of its existence, that the real cause of that mortality is to be derived. It is a fact worthy of notice, that in seven of the cases which recovered, the formation of the abscess was actually completed before a thought of its existence was entertained, and a cure effected by the spontaneous or accidental opening of its cavity, rather than by any preconceived plan of treatment. These facts have led me to consider the subject a very important one, and deserving a careful investigation.

In order to be prepared to comprehend many points in the diagnosis and effects of abscess in this region, it is necessary to remember the anatomical relations of the pharynx; anteriorly, to the tonsils, velum palati, and larynx; laterally, to the internal carotid arteries, internal jugular veins, glosso-pharyngeal, pneumogastric, spinal accessory and hypo-glossal nerves, the superior cervical ganglion of the sympathetic nerve, and some of the deep cervical lymphatics (glandulæ concatenatæ;) and posteriorly, to the five superior cervical vertebræ, with their superjacent muscles, and a few of the deep lymphatic glands, which are prolonged backwards between the pharynx and vertebral column.* These glands are more frequently found behind the pharynx in infants and children than in adults, though their presence is not confined to this period. The situation and arrangement of the petro-pharyngeal, occipito-pharyngeal, and deep cervical aponeuroses, together with the proper fascia of the pharynx, are to be constantly borne in mind, as well as the loose texture of the arcolar tissue, intervening between the pharynx and spine, in which the abscess has its seat.

The occurrence of retro-pharyngeal abscess is confined to no one period of life. It has been met with in an infant in the first month of its existence, and in the adult of sixty years. Of the cases that have been recorded, however, the greater number were observed in children who had not reached the age of ten years. The reason for this more frequent occurrence of abscess at this period it is somewhat difficult to assign; though, perhaps, some propriety may be attached to the suggestion, that, in many instances, it is attributable to a scrofulous diathesis, of an hereditary character. This view receives support from the fact, that in nearly all these patients the disease is traceable either to an inflammation, enlargement and suppuration of the lymphatic glands, behind the pharynx, or to caries of the vertebræ. The irritation and tendency to inflammation, always attendant upon the process of dentition, may also be referred

* *Cloquet's Anatomy*, page 755.

to as influencing in some degree the commencement of suppurative inflammation in this neighborhood.

In the consideration of this subject, two distinct forms of abscess will require attention, the *acute* or *idiopathic* depending upon a local acute inflammation, and the *chronic* or *symptomatic*, consequent upon disease primarily affecting the cervical vertebræ. These two varieties present many points of resemblance, both in their effects upon neighboring organs, and in their surgical treatment; but, at the same time, in their origin, progress, pathological conditions, and medical treatment, there are many and strongly marked distinctions.

### Etiology.

### 1.—*Of Acute Abscess.*

*a. Predisposing Causes.*—The conditions of the system in which abscess is liable to be formed here, are the same which predispose to their formation in other parts of the body, and do not materially differ, whether they contribute to the development of the acute or chronic form. They may be the result of an hereditary scrofulous tendency; for, under the influence of that disease, the lymphatics are particularly liable to inflammation, and that of suppurative character. In a similar manner, a system affected with the poison of syphilis is pre-eminently exposed to the operation of external influences, which a strong and untainted constitution would be able to resist. Long continued habits of intemperance, also, produce an irritable, inflammatory condition of the system, and hence, abscess is not unfrequently in persons addicted to this vice. Difficult dentition has already been referred to as another and an important predisposing cause. To these may also be added that state of the system, and locally, of this region, resulting from various cutaneous diseases, and especially those complicated with soreness of the throat, such as scarlatina, variola, and others.

*β. Exciting Causes.*—One of the most common exciting causes of the formation of acute abscess in this region is exposure to cold and damp air, followed by an inflammation in the pharynx itself, this inflammation proceeding to suppuration, the pus being deposited between the proper pharyngeal fascia, and the muscles of the pharynx lying upon it.

Mr. Fleming, in an article upon this disease, ("*Dublin Journal of Medical Science,*" vol. 17,) asserts his belief, that a very frequent source of its origin is to be looked for in an acute inflammation of the small lymphatics behind the pharynx. He says, "That this affection is, not unfrequently, an acute inflammation of those glands, particularly in childhood, I am strongly disposed to think, and I am confirmed in the opinion even by the history of the very cases which I have adduced. That those glands are only occasionally found in this situation, I admit, and hence, probably, the rare occurrence of this particular form of disease; but that they exist more frequently than is generally imagined, I am equally certain, and I also believe that those affections of the throat termed scrofulous, when engaging the back of the pharynx, and presenting deep ulcerations, are often no more than chronic suppuration and ulceration of them."

In some of the recorded cases, suppurative inflammation was induced by the presence of foreign bodies in the pharynx, such as a bone of a fish, penetrating, or, as in one case, passing entirely through the posterior wall of the pharynx, the bone itself having been found in the cavity of the abscess at the post-mortem examination. M. Mondière has assigned "retrocession of erysipelas of the face," as the cause of an abscess, of this kind, which came under his observation, and M. Prion, of Nantes, has also met with an instance, which he attributes to the same origin. In the earliest of the cases at the New York hospital, to which I have alluded, erysipelas of the face must have coexisted with, and it is possible that it was the cause, direct or indirect, of the abscess. Again, the existence of stricture of the œsophagus, or of rheumatism, has been mentioned as a reason for inflammation, and acute abscess in this region. Sometimes the abscess is developed without any assignable immediate cause, as in the very interesting case reported by Dr. J. Clark, of New Jersey, in the *N. Y. Journal of Medicine,* for July, 1849.

### 2.—*Of Chronic Abscess.*

*a. Predisposing Causes.*—These, as has already been remarked, are of the same character with those of the acute form, and therefore, it is not necessary that they should be here repeated.

β. *Exciting Causes.*—Chronic abscess behind the pharynx is referable, in nearly every instance, to caries, or to tubercular disease of the cervical vertebræ. The process of formation in *psoas* abscess is well understood, and as it is almost precisely identical with that connected with the upper portion of the vertebral column, it will not be advisable at present to enter into any detail with reference to it.

The irritation and subsequent inflammation produced by the presence of a fish-bone, was mentioned as one cause of the acute variety of this disease. This same cause may also be, indirectly, the origin of an abscess, chronic in its formation and general characteristics, by producing, primarily, caries of one of the vertebræ. A case of this kind, the bone piercing the pharynx, and entering the body of one of the vertebræ, is recorded in the *London Lancet*, for June, 1847, page 581.

Having thus briefly alluded to the main circumstances, to which may be ascribed the commencement of this disease, we are prepared to enter upon the consideration of the

### SYMPTOMS.

1. *Of the Acute Abscess.*—The phenomena, which are strictly peculiar to acute pharyngeal abscess, are somewhat equivocal in their nature, and it is principally by symptoms, common to this and the chronic form, that the existence of their true cause would be suspected, and accurately ascertained. The premonitory signs of the disease, like those of nearly all inflammatory affections of the throat, are, an undefined sensation of local uneasiness, stiffness in the back of the neck, accompanied with chilliness, followed, in a greater or less degree, by febrile excitement. These general symptoms are soon succeeded by pain and soreness in the throat, which pain is aggravated during the act of deglutition. Febrile excitement is not, in all cases, well marked, for very frequently the feeling of chilliness is continuous, never leaving the patient entirely, though more severe at one time than at another; a fact peculiar to this form of disease, if associated with the local pain just mentioned, and one that should always lead us to suspect, and endeavor to arrest, if possible, the formation of a purulent deposit in the region of the pharynx. Unfortunately, it is not probable that the aid of the physician would be solicited at so early a period, for in this stage of nearly all the throat diseases, domestic remedies are called in requisition, rather than the advice of an intelligent medical man. In very young children, the commencement of the disease may be attended by convulsions, a fact which receives explanation in the very great preponderance of the nervous system, at this period of life; and the strong tendency to its disturbance by any unnatural source of irritation. Associated with the pain and soreness of the throat, of which I have spoken, there is not unfrequently swelling, œdematous in its character, of the anterior and lateral portions of the neck. Sometimes this tumefaction is very extensive, and will be very liable to occupy the attention to the neglect of the actual source of danger. This symptom is also common to this disease and to œdematous laryngitis, an affection in which it is of exceedingly great importance that an early and accurate diagnosis should be made.

As the disease advances in its course, pain and soreness of the throat are increased, a peculiar fulness about the fauces, and a sensation as of some foreign body arrested at the base of the tongue are experienced, deglutition becomes difficult and painful, the patient complains of excessive thirst, the respiration, at first attended with a slight snuffle, becomes labored, irregular, sometimes hissing, at other times, stertorous or roaring, or accompanied with a gurgling sound, from the passage of air through the viscid mucus, which collects about the fauces; the voice is very much changed, becoming markedly nasal, and resembling that consequent upon cleft palate, a cool perspiration, more or less profuse, appears about the head, the face and surface of the body are pallid, and the pulse sometimes full and forcible, but always quick and very frepuent. If the disease is not recognized, and consequently is allowed to proceed, all these symptoms are rapidly aggravated. The dysphagia becomes very severe, attempts to swallow solid food proving entirely unsuccessful, and even fluids taken into the mouth, being immediately rejected, partly by the mouth, though chiefly by the nostrils. The laboriousness of the breathing is greatly augmented, and interrupted by frequent and convulsive paroxysms of dyspnœa, or of suffocative cough, threatening immediate death. At this stage of the disease, also, in young children, the dyspnœa is liable to produce convulsions, from which the little patients never recover. At other times derangement of the cerebral function, indicated by somnolency, or perhaps coma, is a prominent feature in their case. These paroxysms are induced or rendered more severe, by attempts to swallow, or by assuming the horizontal position, and the patient consequently maintains an erect or partially erect posture. During these attacks of suspended respiration, the face is flushed, and sometimes of a dark leaden hue, the head thrown forcibly backward between the shoulders, the lower maxilla projected forward, the lips livid and cold, the tongue often protruded from the mouth, and the pulse exceedingly rapid, sometimes attaining the height of 130 or 140 beats in a minute. Should the tongue be retracted within the mouth, and the patient requested to protrude it, it is spasmodically thrust out, and returned with considerable difficulty. There is also frequently a coarse mucous râle to be heard along the course of the larynx and trachea.

Upon examining the throat, there *may be* detected more or less congestion of the internal surface of the mouth and pharynx, and when this is the case, there may be also some swelling and redness of the tonsils, and of the epiglottis. Should the seat of the abscess extend· above the level of the glottis, as is the fact, in nearly every instance, a tumefaction of the pharyngeal parietes can be seen, upon which is spread out the velum of the palate. If now the fore-finger be passed into the mouth, back to the posterior wall of the pharynx, a firm, elastic tumor can be distinctly felt, commonly ovoid in shape, situated between the vertebræ and pharynx, pushing forward the latter, and, in many instances, even separating the alæ of the thyroid cartilage of the larynx. This separation can sometimes be detected by an external examination. The tumor may not always be found directly in the median line, and it may involve other organs in the neighborhood, but this does not change the character, though it may influence the severity of the disease, for the results will be the same, and the same plan of treatment will be required. I have spoken of the tumor as conveying an *elastic* feel, rather than one of fluctuation, for the reason that it is almost impossible to obtain, satisfactorily, the sensation of fluctuation in this region, inasmuch as but one finger can be passed down to the swelling.

When the termination of this abscess is fatal, death is almost always the result of asphyxia, produced by compression upon the larynx, though it may be caused by the opening, either spontaneous, or artificial, of the abscess, its contents passing into and deluging the larynx and trachea.

2. *Of the Chronic Abscess.*—The chronic variety of this abscess is almost universally symptomatic of some constitutional disease, traceable, more or less directly, to hereditary or specific taint; and the most frequent of these is caries of the vertebræ. The symptoms which belong exclusively to it are manifested, therefore, principally, during its formation, and are similar, with a few local modifications, to those attendant upon vertebral caries generally. Among the earliest of these are to be noticed more or less stiffness and dull pain about the neck, posteriorly, the pain being increased by the movements of the head, and in some instances, being most severe in the evening and night. Very frequently these phenomena, from the small amount of inconvenience they occasion, are, for a long time, overlooked or neglected, or attributed to other causes than vertebral disease, a neglect which involves consequences of the most serious character to the patient. As the abscess becomes augmented in size, these symptoms become more marked, and are sometimes accompanied by a partial or complete closure of the jaws, a feature of the case, by the influence of which the diagnosis, derived from physical examination, is rendered unsatisfactoy or impossible.

In cases of this kind, the cavity of the abscess is liable to follow a more extended route than is usual in the acute form. Thus, the purulent matter may find its way, downward, through the loose areolar tissue behind the œsophagus, even into the posterior prediastinal space; or again, into the lateral portions of the neck, beneath the deep fascia. In the case reported by Dr. Clark, of New Jersey, to which allusion has been made, the abscess not only existed behind the pharynx, but "extended from the mastoid process down along the course of the sterno-cleido-mastoid muscle of the right side, to the situation of the thyroid gland, which it fully occupied, giving it the appearance of goitre."*

All these symptoms may continue, and the abscess constantly increase in size, for an extended period, before producing any of the phenomena which excite alarm, and demand immediate and active treatment; for it is a well-established law in pathology, that a steady but gradually augmenting pressure may be continued, with impunity, upon organs essential to life, during a long time, no urgent symptoms presenting themselves until the compression has been carried beyond a definite limit.

When the collection of purulent matter has become so great, as to begin to press upon and interfere with the function of important neighboring organs, another class of phenomena is presented. Dysphagia, increasing in severity, is followed by excessive dyspnœa, and nearly all the symptoms of acute idiopathic abscess. These have already been described fully under their appropriate head, and require no repetition. There is one peculiarity, however, which is exhibited in very many cases of the chronic variety, of great importance to recollect. In its later stages, fever, of a low typhoid character, makes its appearance, and, unless it is promptly met and skillfully treated, death will inevitably ensue. The alleviation of the dysphagia, too, by

* *New York Journal of Medicine*, July, 1849.

opening of the chronic abscess, is not always as satisfactory as in the acute form; for, by the long-continued tension, and greatly increased thickness of the posterior wall of the pharynx, its elasticity and contractile power are very much impaired, and the obstruction of the canal continues, nearly as complete as before the opening, the result being, sooner or later, death from defect of nutrition.

**[To be continued.]**

